# Seeing and Feeling Motion: Canonical Computations in Vision and Touch

**DOI:** 10.1371/journal.pbio.1002271

**Published:** 2015-09-29

**Authors:** Christopher C. Pack, Sliman J. Bensmaia

**Affiliations:** 1 Montreal Neurological Institute, McGill University, Montreal, Québec, Canada; 2 Department of Organismal Biology and Anatomy, University of Chicago, Chicago, Illinois, United States of America

## Abstract

While the different sensory modalities are sensitive to different stimulus energies, they are often charged with extracting analogous information about the environment. Neural systems may thus have evolved to implement similar algorithms across modalities to extract behaviorally relevant stimulus information, leading to the notion of a canonical computation. In both vision and touch, information about motion is extracted from a spatiotemporal pattern of activation across a sensory sheet (in the retina and in the skin, respectively), a process that has been extensively studied in both modalities. In this essay, we examine the processing of motion information as it ascends the primate visual and somatosensory neuraxes and conclude that similar computations are implemented in the two sensory systems.

## Abstract 2

A close look at the cortical areas that support vision and touch suggests that the brain uses similar computational strategies to handle different kinds of sensory inputs.

## Introduction

**Fig 1:**
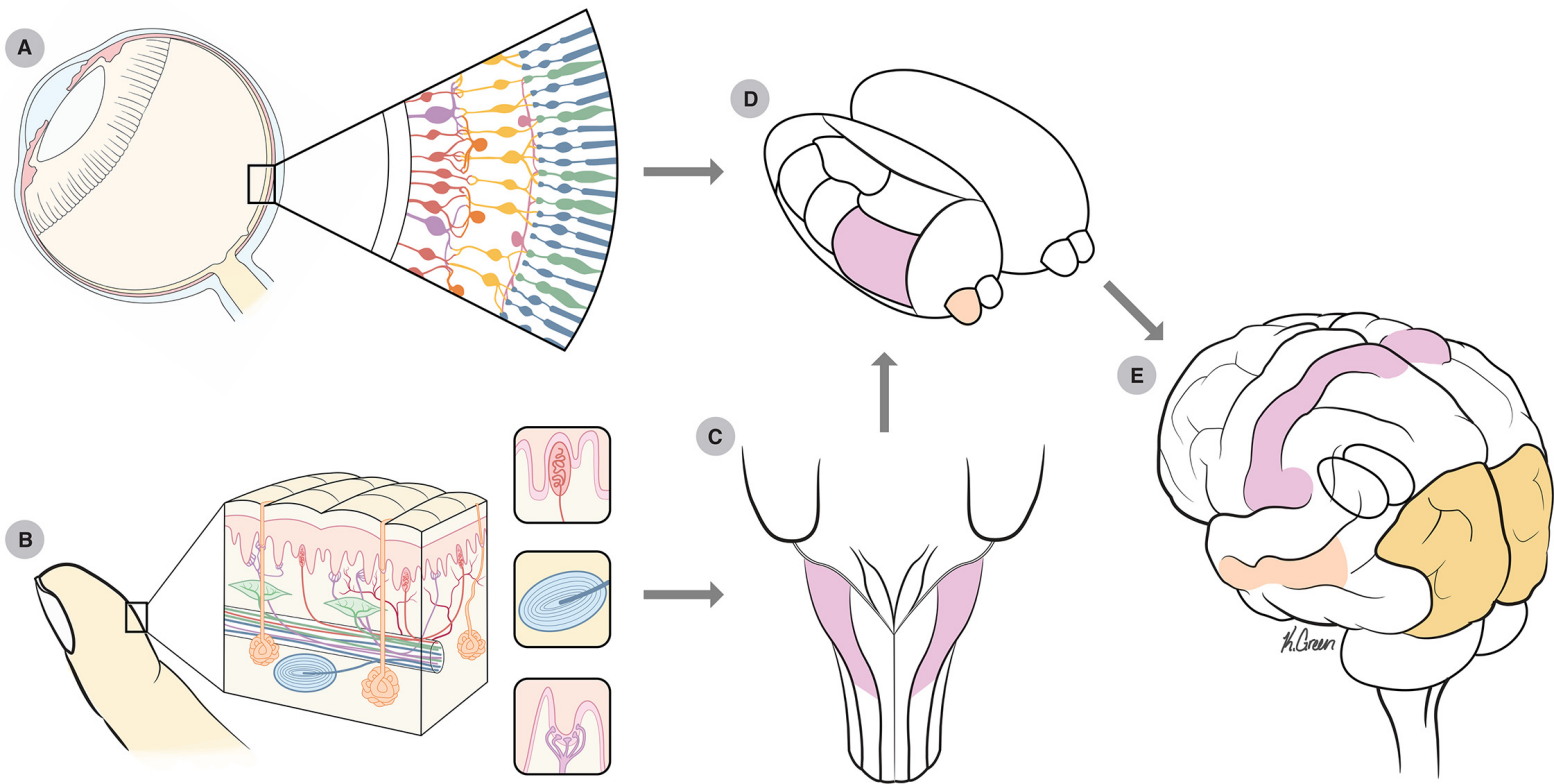
(A) Eye with slice of the retina. (B) Fingertip skin with a representation of a Meissner corpuscle (top), Pacinian corpuscle (middle), and Merkel receptor (bottom). (C) Cuneate nucleus (highlighted in purple). (D) Thalamus. Purple: ventral posterior nucleus; orange: lateral geniculate nucleus. (E) Purple: S1 (including Brodmann’s areas 3b, 1, and 2). Orange: V1. Peach: MT. *Image credit*: *Kenzie Green*.

The nervous systems of humans and other mammals contain sensory receptors that differ in their sensitivities to different categories of stimuli. In touch, mechanoreceptors embedded in the skin respond to physical deformations of the skin; in vision, photoreceptors in the retina respond to light (Fig 1A and 1B). Although the brain modules for processing different types of inputs are largely distinct, the internal organization of these modules is surprisingly similar. In particular, sensory areas exhibit a topographic organization [1], wherein nearby neurons respond to similar stimulus features. This organization is columnar in the sense that, while neuronal response properties differ along a direction parallel to the cortical surface, they tend to be similar along the perpendicular direction [1]. In mammals, columns span the six layers of neocortex, and the connectivity within and between these layers is similar in most brain regions. These commonalities have led to the notion of a canonical circuit [2,3] that implements canonical computations. In this conception, cortical networks devoted to different sensory modalities differ only in the peripheral receptors that provide them with input and are otherwise identical or at least highly similar [4,5].

This is a powerful idea: to the extent that neural circuits perform canonical functions, we may be closer to understanding the brain than we realize. That is, some of the more complex functions performed by sensory systems—face recognition or texture identification, for example—might reflect relatively simple computations, iterated over multiple stages of neural processing in different modalities. Although this idea was proposed long ago on physiological [6,7] and theoretical [8] grounds, there has been little progress in testing it over the ensuing decades [9].

In this essay, we compare sensory processing in vision and touch to assess the degree to which analogous mechanisms are implemented in these modalities to solve analogous problems. To this end, we exploit recent developments that have led to algorithmic descriptions of a key function carried out by both systems, namely the processing of stimulus motion. The development of quantitative models of motion processing has yielded a reasonably clear picture of the computations carried out by the cortex in vision [10,11] and in touch [12]. Moreover, recent advances in statistical modeling have opened up new approaches to identifying and comparing neural computations in high-level sensory structures [13].

We suggest that the brain regions devoted to vision and touch, despite receiving fundamentally different physical inputs, implement many of the same processing strategies. We propose that the identification of canonical computations can be used as a starting point for the development of a quantitative understanding of other brain regions. Such a convergence of ideas has important implications for both basic and applied neuroscience [14].

## Motion Processing in the Periphery and Thalamus

**Fig 2:**
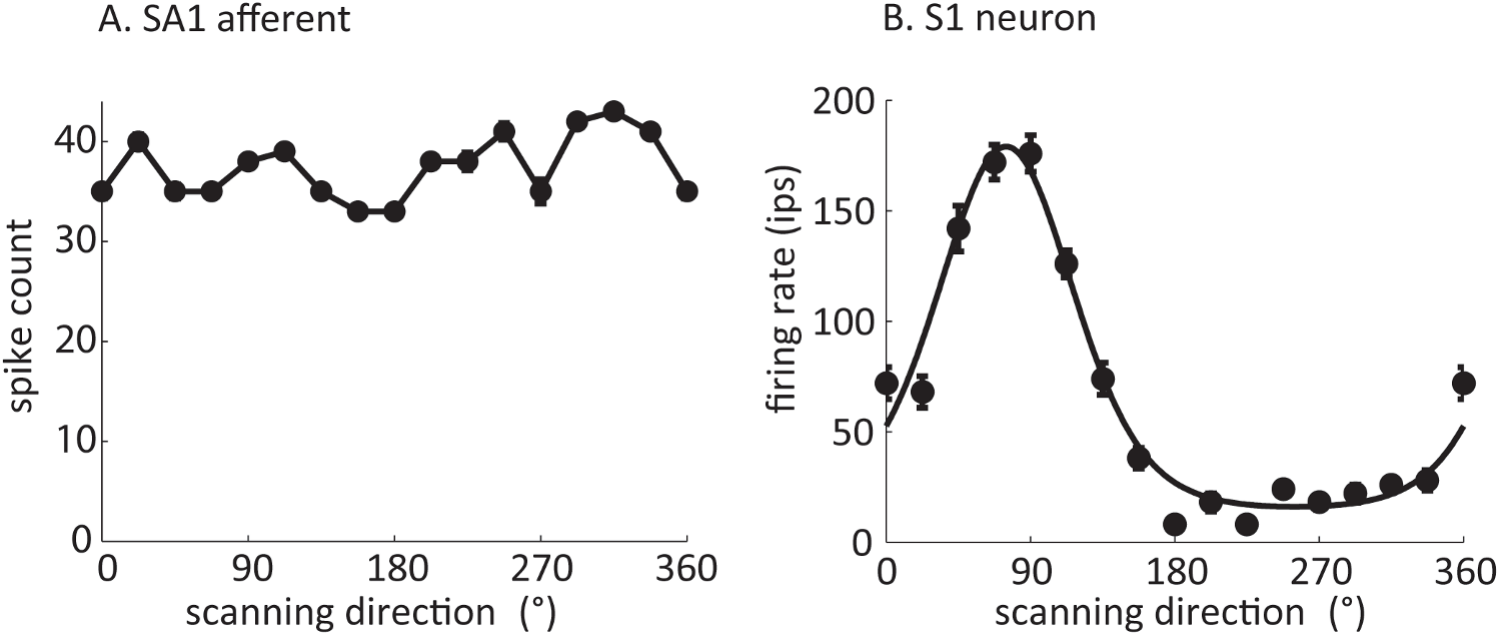
The responses of somatosensory neurons to bars scanned across the skin. (A) The responses of slowly adapting Type 1 (SA1) afferents are relatively insensitive to scanning direction. (B) The responses of a subpopulation of neurons in S1 are strongly tuned for scanning direction [19].

A moving object is one that changes position over time within some reference frame. When the reference frame is a receptor surface, the job of the nervous system is to estimate the object’s velocity from the outputs of peripheral receptors. In species such as mice and rabbits, strong velocity selectivity is found in the outputs of individual neurons in the sensory periphery [15]. In contrast, while some direction tuning is observed at the visual [16] and somatosensory periphery [17,18] under some circumstances, it tends to be much weaker than that observed in cortex (Fig 2) [16,19]. This suggests that, in primates, estimates of stimulus velocity are computed more centrally from peripheral signals.

In the primate retina, at least two populations of neurons contribute to motion processing (Table 1). Magnocellular neurons have relatively large receptive fields and respond best to transient stimulus events [20], whereas parvocellular neurons have smaller receptive fields and respond well to slow motion [20]. Similarly, rapidly adapting (RA) and Pacinian (PC) afferents in touch (which innervate Meissner and Pacinian corpuscles, respectively) have larger receptive fields (RFs) and respond to rapid skin deflections, whereas slowly adapting Type 1 (SA1) afferents (associated with Merkel receptors) have small RFs and respond well to slow-moving or stationary stimuli (Fig 1A) [21–23]. Thus, individual afferent classes in both vision and touch exhibit different selectivities for temporal structure in the stimulus. These neurons also have receptive fields that are quite small (with the exception of PC afferents in touch), indicating that they can also signal the position of a stimulus with high accuracy. Selectivity for the temporal and spatial structure of the stimulus is, however, insufficient to establish velocity selectivity, which entails a neural preference for motion in some directions over others, as well as tuning for a specific range of speeds.

**Table 1:** Types of peripheral receptors that contribute to motion processing.

	Parvo Cells and SA1 Afferents	Magno Cells and RA and PC Afferents
Receptive fields	Small	Larger
Activation thresholds	High	Low
Most sensitivity	Most sensitive to slow motion	Most sensitive to fast motion
Rate-intensity function	Approximately linear	Saturating

Inputs from visual and somatosensory afferents are relayed to cortex via thalamic nuclei. In touch, there is an intervening synapse in the cuneate nucleus, where cutaneous signals may be processed to more closely match those of retinal ganglion cells, a hypothesis that has yet to be formally tested (Fig 1C and 1D). In the visual system, the lateral geniculate nucleus (LGN) provides the main relay from the periphery to the primary sensory cortex, while the analogous structure for touch is the ventral posterior nucleus (VPN). Although modest directional biases have been observed in the responses of individual LGN neurons to visual motion [24], strong direction selectivity is effectively absent in this thalamic nucleus. The same is likely true for VPN neurons, although this has not been systematically investigated.

## Spatiotemporal Processing of Inputs in Primary Visual and Somatosensory Cortices

In primates, robust neuronal selectivity for the direction of visual motion first appears in the primary visual cortex (area V1, Fig 1E). Although different studies have applied different criteria for classifying a cell as direction selective, the typical finding is that roughly 15%–30% of V1 neurons exhibit this property [25–27]. Similarly, robust direction selectivity is found in about 30% of neurons in Brodmann’s area 3b (Fig 1E, Fig 2B) [19], which, along with area 3a, forms the primary somatosensory cortex proper.

**Fig 3:**
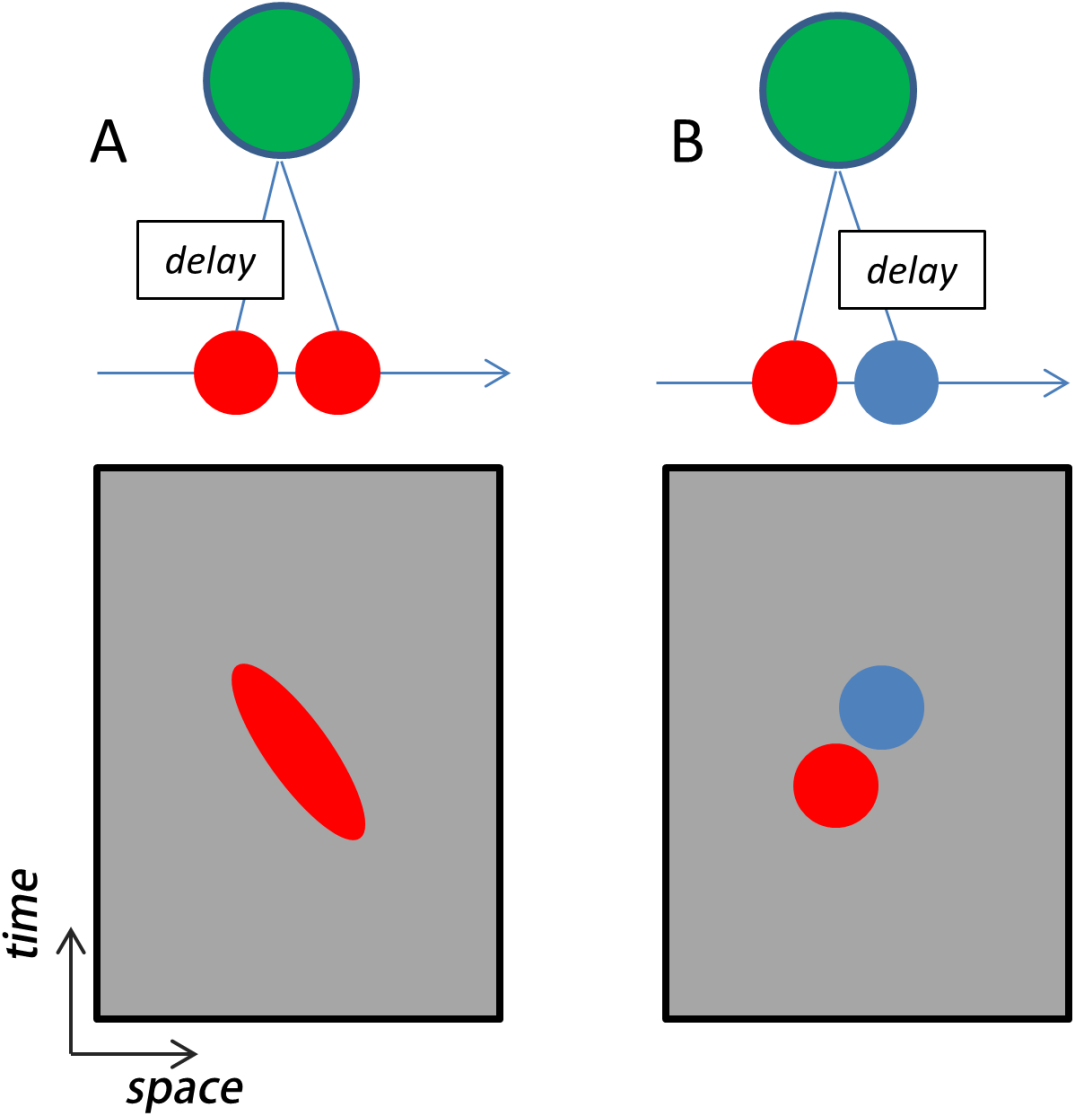
Models of elementary motion selectivity. (A) The space-time slant model. In this model, direction selectivity is due to the spatiotemporal preferences of the excitatory inputs (red). That is, the outputs of neurons with different receptive field positions and response latencies combine to produce a stronger response for rightward than for leftward motion. (B) The inhibitory “veto” model. In this conception, a moving stimulus activates a suppressive input (blue) if it passes through a certain position in space. Because this suppression is delayed, it arrives simultaneously with the input from an excitatory input (red). The suppression and excitation cancel out, effectively yielding no response to motion in the nonpreferred (here, leftward) direction.

Many models have been proposed to account for the emergence of direction selectivity in the primary visual cortex. From a theoretical perspective, the problem is to integrate the outputs of thalamic neurons in such a way as to derive selectivity for motion direction and speed. This approach is conceptually identical to that of the Hubel and Wiesel model of orientation selectivity, with velocity simply being orientation in space-time (Fig 3) [28]. Examination of the structure of spatiotemporal receptive fields in visual and somatosensory cortices thus provides a critical comparison of computation in the two modalities.

Early studies in the visual system revealed two mechanisms that were consistently associated with direction selectivity. The first is a facilitation of a neuron’s response to a stimulus at one spatial position by the previous appearance of another stimulus at a nearby position (Fig 3A). In this scenario, direction selectivity results from an interaction between two or more excitatory inputs, and the preferred direction is determined by the relative positions of the receptive fields of these inputs [28]. The second is a suppression of the response to a stimulus at one position by a stimulus at a different position (Fig 3B). In this case, direction selectivity results from a synaptic mechanism that effectively vetoes responses in the nonpreferred direction [15].

Excitatory receptive field interactions can arise simply from afferent inputs that exhibit different response latencies at different spatial positions [29]. Specifically, integration over the outputs of afferents with suitable spatial positions and response latencies can yield receptive fields that exhibit orientation in space-time (Fig 3A), the angle of which reflects the preferred velocity [28]. Receptive fields with excitatory space-time orientation are found in both V1 [30] and S1 [31].

Evidence for the suppressive mechanism in primate V1 comes from physiological studies that show that the response to a flashed stimulus is reduced when it is preceded by another flashed stimulus at a spatially offset location [32]. The mechanism responsible for this property is a suppressive input that arrives at the neuron with some delay relative to the excitatory inputs. The spatial arrangement of stimuli that generate this interaction is generally consistent with the neuron’s preferred direction. Similarly, direction selectivity in many S1 neurons also relies on a lagged and spatially offset suppressive component [31].

In summary, the emergence of direction selectivity in primary visual and somatosensory cortices involves a combination of excitatory and suppressive mechanisms. These are instantiated through integration of thalamic inputs with specific spatial and temporal selectivities. It is important to note that there is nothing inevitable about this result: direction selectivity could be computed in the sensory periphery [15] or by other mechanisms that would yield spatiotemporal receptive fields different from those observed [33].

## Hierarchical Motion Processing: Beyond Primary Sensory Cortex

Given the strong selectivity for stimulus orientation in V1 and S1, experimenters typically study motion processing with oriented stimuli that move across the receptive field. While many V1 and S1 neurons exhibit strong direction selectivity to this kind of stimulus, their direction selectivity for stimuli that contain multiple orientations, such as random dot fields [19,34], is much weaker. Both S1 and V1 send projections to cortical areas that are either specialized for motion processing or contain subpopulations of neurons that are, namely the middle temporal (MT or V5) area in vision [35] and Brodmann’s area 1 in touch [19,36]. In contrast to their counterparts in earlier areas, neurons in MT and area 1 exhibit strong direction selectivity for random dot fields [19,27]. This selectivity is thought to arise via integration of inputs from primary cortical neurons with many different orientation preferences [37].

**Fig 4:**
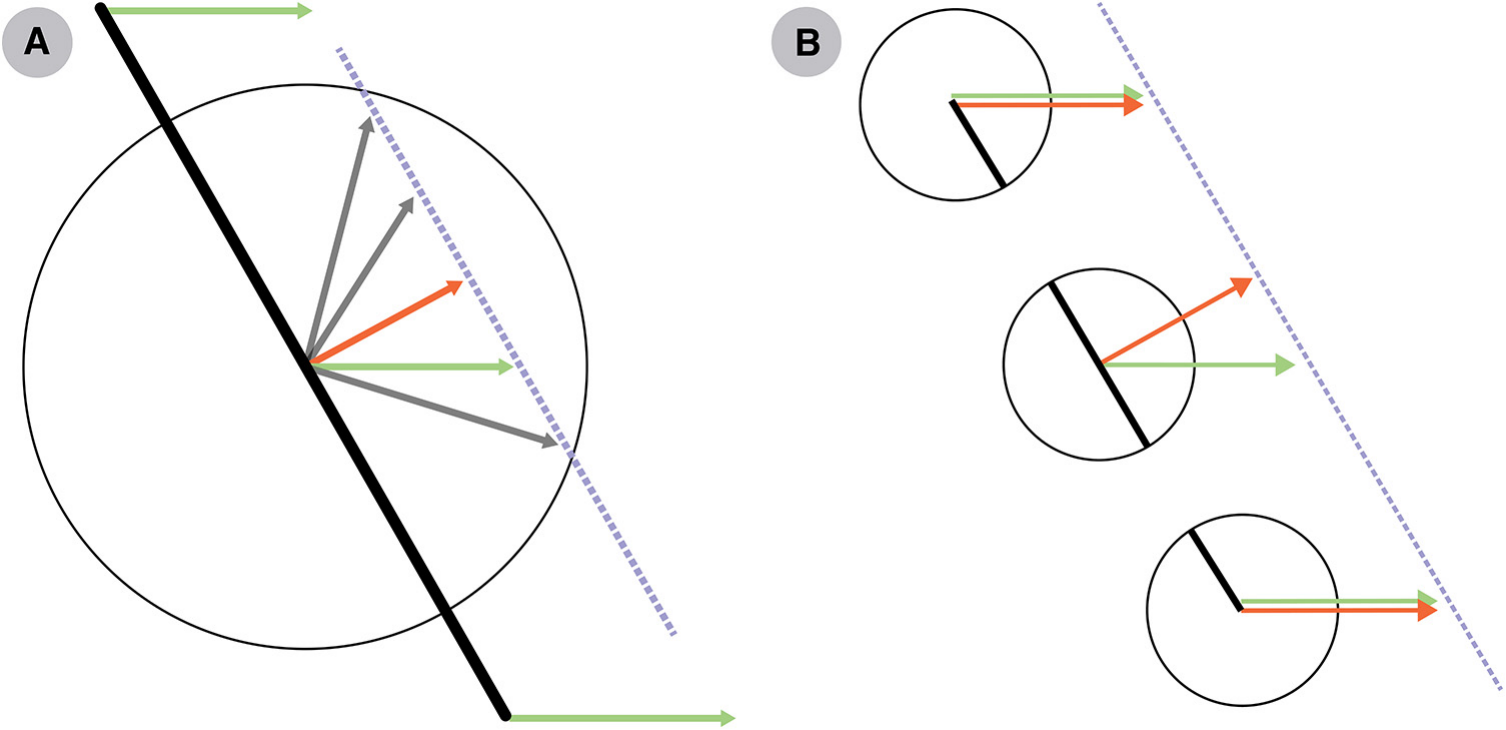
Limitations on motion processing by neurons with small receptive fields. (A) A neuron stimulated by an edge drifting through its receptive field, denoted by the circle, can only estimate the component of motion perpendicular to the edge orientation (red arrow), which is not generally the same as the actual direction (green arrows). (B) Small receptive fields compute velocity estimates (orange arrows) that depend on local stimulus structure. An end-stopped receptive field performs feature detection by responding to the end points (top and bottom) of a contour, which provide accurate estimates (green arrows). On the other hand, these receptive fields do not respond to continuous contours (center circle).

In addition to integrating over orientations, neurons in both MT and area 1 integrate across space. Indeed, individual RFs in area V1 cover a tiny fraction of the visual field [25]; similarly, the majority of RFs in area 3b are smaller than 40 mm^2^, so most of them cover a small fraction of a finger pad [38]. Such small receptive fields can be problematic for motion processing, because a small field of view does not necessarily permit reliable estimates of velocity for larger objects (Fig 4A). In contrast, neurons in both MT and area 1 have relatively larger RFs [39,40].

Consistent with this hierarchical organization of sensory cortices is the view that motion processing requires at least two stages [41]. Theoretically, there are many ways to formulate this two-stage process [10]. One class of models hypothesizes that object velocity is explicitly represented only at the second stage, while a second class of models hypothesizes that an initial estimate is obtained at the first stage, based on local features (Fig 4B). The two models are not mutually exclusive, and a combination of the two mechanisms would likely yield more robust and precise estimates of object velocity [10].

Evidence for the first class of models comes from the observation that some neurons in MT and area 1 appear to estimate object velocity in a manner that is independent of the spatiotemporal structure of the stimulus [19,42], in contrast to earlier stages of visual or tactile processing. For example, in touch, scanned bars yield direction-selective responses in area 3b, while random dot fields do not [19]. A similar phenomenon is obtained with plaid stimuli, which contain edges moving in two or more directions. When the orientations and speeds of the edges are chosen properly, the stimulus appears as one pattern moving in a single direction. While individual neurons in areas MT and area 1 exhibit selectivity for this pattern motion [19,43,44], such selectivity is generally lacking in earlier areas [19,43].

In the second class of models, feature extraction in the primary sensory cortex is tailored to facilitate accurate velocity estimation. Theoretically [45], the most informative features are those that contain multiple orientations in small image regions, for example, corners and line intersections. Because these features are defined locally, they can be detected with small receptive fields that exhibit more complex selectivity than a preference for a single orientation. Such selectivity was first noted in V1 by Hubel and Wiesel [46], who found evidence for neurons that responded best to short line segments; the responses of these “end-stopped” neurons were suppressed by extended edges. Similarly, a subpopulation of neurons in area 3b exhibit a receptive field structure that would in principle yield end-stopped responses, although they have not been tested specifically for this property [31,47]. Pack et al. [48] showed that end-stopped neurons could encode motion direction in a manner that was to some degree independent of the spatial configuration of the stimulus. With this mechanism in place, the responses of neurons in MT and area 1 can often be predicted based on a simple average of their inputs from V1 and area 3b, respectively, assuming that input from end-stopped neurons is weighted more heavily than that from motion-selective edge detectors [36,49]. There is thus indirect evidence to support the idea that visual and tactile motion processing benefits from more complex feature extraction at an early stage, and this idea has been incorporated into more recent computational models [36,50–53].

These considerations highlight two key computations that are shared between vision and touch. The first is hierarchical processing: stimulus velocity is computed in stages, and the algorithmic details of the computations at each stage are quite similar across the two modalities. The second is feature detection: the extraction of specific features at one stage facilitates computation at the next stage. Again, there is nothing inevitable about these similarities: theoretical work has shown that alternative approaches can compute velocity very well [37].

## Velocity Perception in Vision and Touch

Given the similarity and sophistication of motion processing in higher-order regions of visual and somatosensory cortex, one might ask whether these neuronal populations lead to similar perceptual experiences of motion and can account for observers’ perceptual reports of motion direction. This question can be addressed with plaid stimuli, for which the perceived direction depends heavily on the precise composition of the stimulus, namely, the respective motion directions of the component gratings. Psychophysically, the perceived direction depends heavily on stimulus composition, and this dependence is similar for tactile and visual plaids [54]. Furthermore, paired psychophysical experiments (in humans) and neurophysiological experiments (in monkeys) have shown that the responses of neurons in higher-level cortical areas account for the perceived direction of plaids across a wide range of conditions in both vision [43,55] and touch [36].

Visual and tactile velocity perception has also been studied with random-dot motion stimuli that are corrupted with random noise. As more noise is added to the stimulus, the task becomes more difficult, and one can then examine the conditions under which neural processing and the perception of directed motion begin to break down. Work in area MT has shown that the sensitivity of individual neurons to visual motion is similar to that of observers [56], suggesting that perceptual decisions about visual motion can be driven by the outputs of a small number of MT neurons. Similarly, in touch, the mean sensitivity to changes in direction of individual direction-selective neurons in area 1 matches that of human observers across all tested conditions [19].

## Why Are Visual and Tactile Processing So Similar?

In vision and touch, stimulus information is extracted from a spatiotemporal pattern of activation across a sensory sheet, in the retina and the skin, respectively. The peripheral signals from the two systems are analogous, with magnocellular retinal ganglion cells corresponding to RA (and perhaps PC) fibers and parvocellular cells to SA1 fibers. At the earliest stage of cortical processing (V1, area 3b), visual and tactile motion signals are extracted by neurons with a specific spatiotemporal receptive field structure in such a way that their responses are highly dependent on the spatial properties of the stimulus. In the next hierarchical stage, motion representations are relatively independent of stimulus shape, due in part to the extraction of informative features in the primary sensory cortex. The receptive field structure, hierarchical sequence, and feature detection computations appear to be similar across modalities. One might ask, then, why motion processing in vision and touch is so similar.

One possibility is that the statistics of the stimuli that impinge on both systems in everyday life are similar. Indeed, objects have edges that move across the sensory sheet with a distribution of velocities that are approximately analogous in both modalities. As such, it is possible that analogous mechanisms evolved independently following principles of efficient coding [57]. A related possibility is that both systems evolved from a common receptor type; indeed, there is evidence that basic visual circuitry has been conserved across species, over millions of years [58]. In addition, visual motion computations are highly similar between insects and primates [13,29,59], to the extent that models of motion processing in the beetle predict human motion perception with remarkable fidelity [29].

Another possibility is that a fundamental principle that guided the evolution of these two sensory systems is that the resulting sensory representations be expressed in a common language that allows these to be integrated and, when necessary, mutually recalibrated. The integration of visual and tactile representations is well documented, including in motion processing. Indeed, the visual perception of motion has been shown to interact with its tactile counterparts in a variety of behavioral contexts [60–62], which has been interpreted as evidence that these motion representations converge somewhere along the neuraxis [63].

The notion of canonical computation likely extends beyond the motion domain. In fact, the computations described above are similar to those involved in the extraction of shape information, which is also analogous in vision and touch [64,65], and strong analogies between vision and audition can be drawn as well [5]. This convergence of ideas holds great promise for future neuroscience investigations: if the bewildering complexity of sensory cortex can be reduced to a few canonical computations, we can narrow our search for candidate mechanisms. The search for canonical computations may thus dovetail with that for canonical neural circuits and lead to a more integrated view of nervous system function.

## Abbreviations

LGN: lateral geniculate nucleus
MT: middle temporal
PC: Pacinian corpuscle
RA: rapidly adapting
RF: receptive field
SA1: slowly adapting Type 1
VPN: ventral posterior nucleus
